# Poisoning by Belladonna

**Published:** 1859-01

**Authors:** B. F. Schneck

**Affiliations:** Lebanon, Penn.


ARTICLE VII.

### POISONING BY BELLADONNA.

BY B. F. SCHNECK, M. D., OF LEBANON, PENN.

I have been in the habit for several years past of treating many cases of hooping cough with the following prescription, and have been invariably well pleased with the result:

℞ Extr. belladonnæ, grs. xij.
Syr. ipecac., ℥j.

Tere et misce sig., 10 to 30 drops three or four times a day.

A little son of H. R., aged 4 years, convalescent from the disease under this treatment, had just been ordered half an ounce more of the mixture, as being sufficient to remove what little cough there yet remained. Of this vial he had taken but one dose, when, his mother being engaged in another room for fully half an hour, the child drank the whole half ounce, save what little of the syrup, owing to its thickness, adhered to the inside of the glass. When she returned she remarked his odd behavior, for which she could not at first account. After the lapse of nearly an hour, however, finding the empty vial, and now remembering my caution as to the danger of the remedy in over-doses, she sent me a very emphatic summons, which, it is needless to say, I attended to with more than obstetric haste.

I found the child completely belladonnized, and the worst not yet come. There was a disposition to drink constantly, indicating great dryness of the mouth and throat; complete loss of sight, with intense injection of the eyes, and great flushing of the face. He appeared to hear morbid sounds, as indicated by his suddenly starting up into a listening attitude and looking round. The next moment he fell into a short sleep, from which he soon started up affrighted. The pulse was now very full, feverish and frequent; there was an eruption of a scarlet color over the entire surface, and there occurred occasional efforts at retching and vomiting. Now also visual illusions began to appear, manifested by the child's fancying that he saw objects in his vicinity, and reaching towards them accordingly. Delirium now also came on, associated apparently with pleasant or amusing ideas, for the child would smile and then suddenly burst out into gleesome laughter, accompanied by odd gestures such as children often make in their frolics in health. On standing him out on the floor he could not walk, but staggered, with the body bent forward and stooping, and the arms thrown out gropingly as a blind man would feel his way, and unless held he soon fell down. He now began to be comatose; the surface looked purplish, and the extremities became cool. The treatment, unfortunately delayed for more than an hour for the reason above given, consisted in cold effusions uninterruptedly to the head, and emetics of ipecacuanha and sulph. zinc, which, however, were administered with the greatest difficulty. Ten grains of each were given every twenty minutes, but so insensible was the stomach that upwards of two hours elapsed before free emesis could be induced, and then only by tickling the fauces with a feather. When, at last, vomiting occurred, the child was unconscious, the poison having had a fair time to be absorbed, and the case looked hopeless. A stimulating injection, however, which had been given at the beginning, now fortunately began to operate, and very soon, as if by magic, the symptoms improved. A large dose of calomel with castor oil were now given, and in six hours afterwards the child was out of danger.

The extract was Tilden's, and the quantity swallowed was six grains. It is singular that the half ounce of syr. ipecac. should not of itself have induced vomiting. Probably the nervous sensibility of the stomach was at once overpowered by the quantity of the narcotic taken. The recovery of a child, especially after such a dose, is remarkable; inasmuch as two grains have been known to produce alarming symptoms in an adult.

